# The LEM-ESCRT toolkit: Repair and maintenance of the nucleus

**DOI:** 10.3389/fcell.2022.989217

**Published:** 2022-09-12

**Authors:** Sapan Borah, Karthigeyan Dhanasekaran, Santosh Kumar

**Affiliations:** ^1^ National Institute of Immunohaematology, Mumbai, Maharashtra, India; ^2^ Regional Centre for Biotechnology, NCR Biotech Science Cluster, Faridabad, Haryana, India; ^3^ National Centre for Cell Science, Pune, Maharashtra, India

**Keywords:** NPC, lem, ESCRT, nuclear envelope, NEBD, NUP

## Abstract

The eukaryotic genome is enclosed in a nuclear envelope that protects it from potentially damaging cellular activities and physically segregates transcription and translation.Transport across the NE is highly regulated and occurs primarily *via* the macromolecular nuclear pore complexes.Loss of nuclear compartmentalization due to defects in NPC function and NE integrity are tied to neurological and ageing disorders like Alzheimer’s, viral pathogenesis, immune disorders, and cancer progression.Recent work implicates inner-nuclear membrane proteins of the conserved LEM domain family and the ESCRT machinery in NE reformation during cell division and NE repair upon rupture in migrating cancer cells, and generating seals over defective NPCs. In this review, we discuss the recent in-roads made into defining the molecular mechanisms and biochemical networks engaged by LEM and many other integral inner nuclear membrane proteins to preserve the nuclear barrier.

## Introduction

**FIGURE 1:**
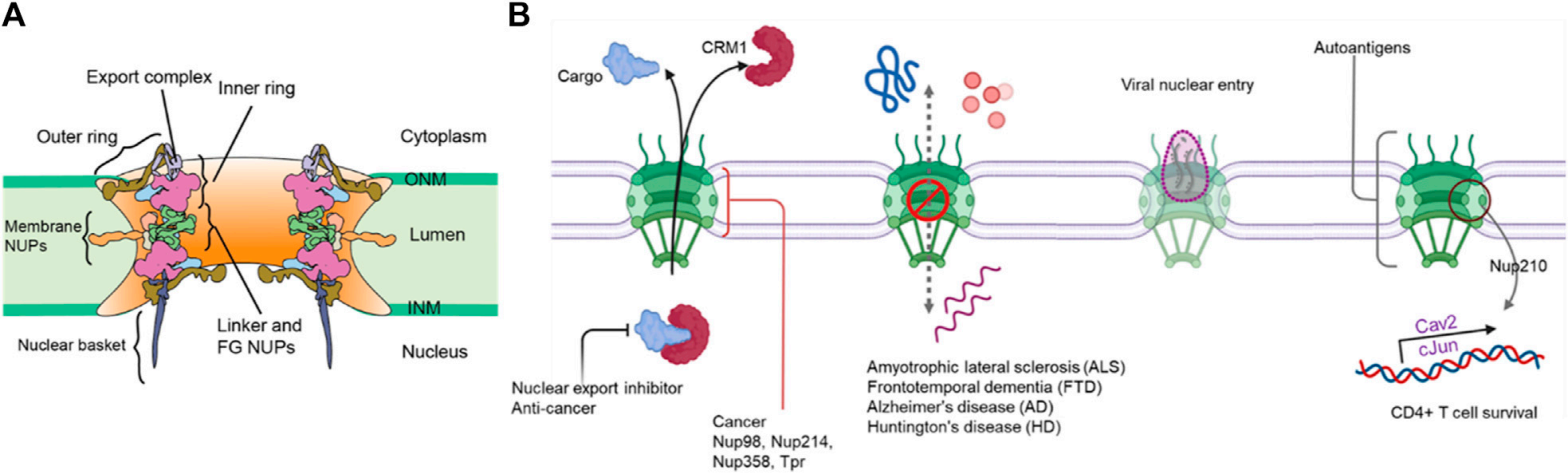
**(A)** General structure of the NPC **(B)** A schematic of NPC-related diseases.

The eukaryotic genome is enclosed in a double membraned nucleus which serves to compartmentalize the genetic material and segregates processes like DNA replication and transcription processes from the cytosol. The nuclear envelope is continuous with the endoplasmic reticulum and is composed of two membranes, inner (INM) and outer nuclear membranes (ONM), separated by a lumen (Ungricht and Kutay, 2017; Hampoelz et al., 2019). Transport across the nuclear envelope barrier is highly regulated and occurs primarily through the nuclear pore complex (NPC). NPCs are macromolecular complexes consisting of ∼550 subunits in yeasts and up to ∼1,000 protein subunits in humans and composed of ∼30 nucleoporin (nups) subunits in multiple copies (Kim et al., 2018; Hampoelz et al., 2019; Lin and Hoelz, 2019). The nups are assembled in subcomplexes that are arranged in concentric rings and radially in an eight-fold symmetry to form the structural scaffold of the NPC (Wente and Rout, 2010; Hampoelz et al., 2019; Paci et al., 2021).The cytoplasmic face is coated with cytoplasmic filaments and an mRNA export complex, while the nucleoplasmic face has a nuclear basket. The central channel is filled with a matrix of disordered proteins containing FG amino acids in repetitive motifs (FG-nups) anchored to the NPC scaffold. The FG-repeats in the central channel form a diffusion barrier limiting the transport of macromolecules and also serves as the binding site for nuclear transport receptors for selective transport across the NPC (Figure 1A) (Wente and Rout, 2010; Paci et al., 2021). Proper assembly of several nups to form these NPCs is crucial since the defects in NPC function and structure are often tied to neurological and ageing disorders like Alzheimer’s, immune disorders, tumorigenesis, cancer progression and also in establishing certain viral infections (Figure 1B) (Sakuma and D’Angelo, 2017). In addition, deterioration of NPC function and assembly upon aging is directly associated with defects in nuclear envelope integrity and transport (D’Angelo et al., 2009; Rempel et al., 2019).

Given the complexity and size of the NPC, cellular machinery must ensure that individual nups are properly assembled to avoid any loss of function and, potentially, nuclear envelope integrity per se. As of now, four classes of proteins have been implicated in NPC assembly; 1) LEM (Lap2-emerin-Man1) and ESCRT (endosomal sorting complexes required for transport) proteins, 2) lipid metabolism-related proteins, 3) membrane bending proteins, and 4) the scaffold nups (Walther et al., 2003; Scarcelli et al., 2007; Dawson et al., 2009; Makio et al., 2009; Webster et al., 2014, Webster et al., 2016a; Lone et al., 2015; Souquet et al., 2018; Zhang et al., 2018; Bilir et al., 2019; Thaller et al., 2019b).

It was recently discovered that the Man1/Heh2 associates with NPCs in *Saccharomyces cerevisiae* and *Schizosaccharomyces pombe,* and this aids in sensing the NPC scaffold assembly state (Borah et al., 2021), which has been proposed to mediate the NPC quality control in conjunction with the Vps4/ESCRTIII machinery (Webster et al., 2014; Webster and Lusk, 2016).

**FIGURE 2:**
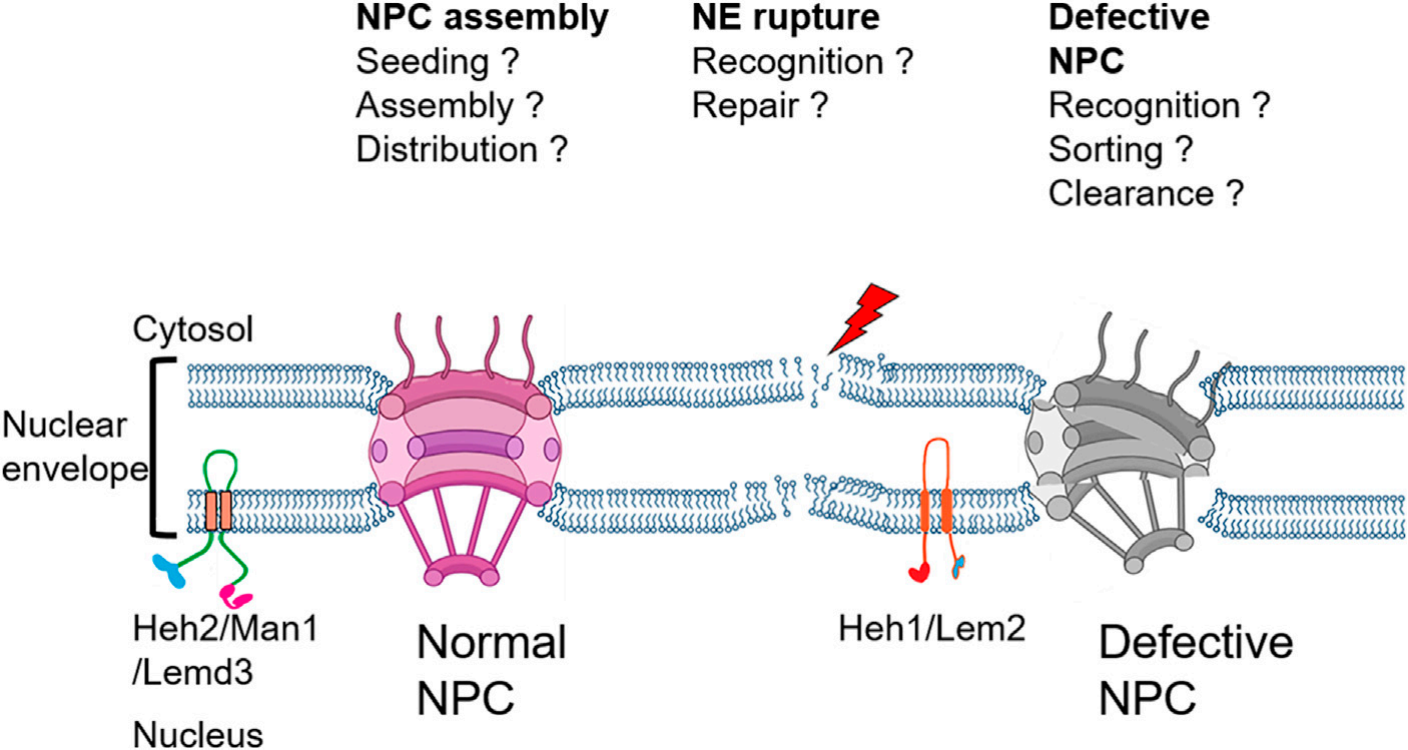
Schematic of the nuclear envelope listing key questions addressed in this review.

In this review, we discuss the cellular mechanisms implicated in the maintenance of the nuclear envelope compartmentalization with a special focus on the recent studies dissecting the role of INM proteins pertaining to the LEM family in recruiting the ESCRT machinery (Figure 2) for a successful nuclear envelope repair.

## Nuclear envelope proteins

Although the structure and function of the nuclear envelope and the nuclear pore complex are highly conserved across eukaryotes, the repertoire of nuclear envelope-associated proteins is known to be different. The presence of lamin proteins shows the most prominent difference. Lamins are type-V intermediate filaments that form a filamentous framework under the inner nuclear membrane and provide structural support to the nucleus and are also closely associated with the NPC at the nuclear envelope (Aaronson and Blobel, 1975; Dechat et al., 2010). Lamins are present in most metazoans but are absent in plants and unicellular eukaryotes. Lamins are categorized into two types, Lamin A and B. Type A-lamins have two isoforms, lamin A and C, which are produced from an event of alternate splicing of the *LMNA* gene.There are two major types of B type-lamins, isoforms Lamin B1 and B2. The expression of Lamin type differs in various metazoans; however most of them express a type of lamin-B (Xie and Burke, 2017).

Super-resolution imaging in LMNA−/− mouse fibroblasts showed an abnormally clustered distribution of NPCs compared to a uniformly spread pattern in the wild type, suggesting that NPCs associate physically with Lamin A (Xie et al., 2016; Xie and Burke, 2017). The NPC component, Tpr, most likely mediates the association of NPC with Lamin C. Proximity labelling studies with Bir-A: LMNA and Bir-A: LMNC showed preferential association of the NPC component Tpr to Bir-A-La-C (Xie et al., 2016). This is also reflected in the sequential dSTORM and PALM based super-resolution imaging, revealing the preferential association of NPCs to the LmnC network (Xie and Burke, 2017). Further, the physical association of NPCs with the Lamin network was corroborated by the high degree of NPC mobility observed in yeasts, which inherently lack Lamins (Belgareh and Doye, 1997; Bucci and Wente, 1997). The Lamin framework remains intact through a cell’s lifetime except at the onset of mitosis. Phosphorylation of Lamins by cyclin-dependent kinase (Cdk) 1 and protein kinase C (PKC) triggers its disassembly (Gerace and Blobel, 1980; Dessev et al., 1988, 1991; Dessev and Goldman, 1988; Peter et al., 1990; Collas, 1999). The lamina is reassembled during telophase to early G1 transition which is initiated by the Lamin dephosphorylation (Thompson et al., 1997). In addition to the chromatin, lamins are associated with the transmembrane proteins of the inner nuclear membrane and together constitute the nuclear lamina. Hence, mutations in both the lamins and the INM proteins are often associated with tissue-specific neuromuscular and developmental diseases that are collectively referred to as laminopathies. (Table 1).

**TABLE 1:** Well-studied inner nuclear membrane proteins enlisting the associated human diseases reported in mutant forms and the related references.

INM protein	Disease	Genetic aberrations and other details	Clinical manifestation	References
MAN1/ LEMD3	Autoantibodies	80, 58, and 40 kDa MAN antigens	Collagen vascular disease	Paulin-Levasseur et al., (1996); Lin et al., (2000)
	Buschke-Ollendorff Syndrome	Y441X, Exon 1, c.332_333insTC Frame-shift, premature stop codons W855X, R655X	Connective tissue naevi	Mumm et al., (2007); Zhang et al., (2009)
	Melorheostosis With Osteopoikilosis	c.1609C ˃ T	Sclerosing bone dysplasias	Hellemans et al. (2004)
LEM2	Cataract 46, A Juvenile-Onset, with or without arrhythmic cardiomyopathy	p.T38G	A type of cataract resulting in the opacification of the crystalline lens of the eye	Boone et al. (2016)
	Marbach-Rustad Progeroid Syndrome	p.S479F	Progeria-like facial appearance, growth retardation, hypoplastic jaws, supernumerary teeth	Marbach et al. (2019)
Emerin	Emery-Dreifuss Muscular Dystrophy (EDMD)	c.153_154insC in exon 2, c.359_362delCAGT in exon 4	Degenerative myopathy, muscle atrophy, contractures, muscle weakness, and heart disease	Ura et al. (2007)
SUN1	EDMD	p.G68D, p.G338S, p.W377C		Meinke et al. (2014)
SUN2	EDMD	p.R620C, p.E438D		
LAP1	Muscular dystrophy and progressive Dystonia with cerebellar atrophy	c.961C > T resulting in a stop codon	Cataract extraction, microphthalmia, severe truncal hypotonia, severe progressive neurological impairment	Fichtman et al., (2019)
Lamin B receptor (LBR)	Greenberg dysplasia, Pelger-Huet anomaly with mild skeletal anomalies	p.N547D, p.R583Q, premature stop codons p.Y468TfsX475, p.V11EfsX24, c.1599-1605TCTTCTA ˃ CTAGAAG	Bone abnormalities in the developing fetus, lethal *in utero*,	Waterham et al., (2003); Konstantinidou et al., (2008); Clayton et al., (2010); Tsai et al., (2016); Turner and Schlieker, (2016)
LAP2α	Dilated cardiomyopathy	p.R690C	Reduced left ventricular ejection fraction, higher left ventricular end-diastolic diameter	Taylor et al., (2005)
LAP2β	Cancers	Overexpressed in gastric cancer tissues	Digestive tract cancer	Kim et al. (2012)
LMNA (LaminA/C)	Hutchinson-Gilford Progeria Syndrome	c.1824C ˃ T, p.G608G, p.G608S, p.E145K	Premature aging, Alopecia, subcutaneous fat loss, atherosclerosis, musculoskeletal degeneration, hearing loss	De Sandre-Giovannoli et al., (2003); Eriksson et al., (2003); Coppedè, (2021)

## Inner nuclear membrane proteins

The nuclear envelope is rich in proteins, and mass spectrometric analysis of nuclear envelope fractions from rat liver, skeletal muscle, and human leukocytes have identified several tissue-specific transmembrane proteins at the nuclear envelope (Korfali et al., 2010, 2012; Wilkie et al., 2011). A split-GFP screen has identified ∼400 transmembrane proteins at the inner nuclear membrane (Smoyer et al., 2016). Of these, the well-studied INM proteins are the LAP2-Emerin-Man1 (LEM)-domain proteins, MAN1, LEM2, and lamina-associated polypeptide 2 (LAP2) and emerin, SUN-domain proteins SUN1 and SUN2, lamin B receptor (LBR) and NET5/SAMP (Table 1). Table 1 gives an outline of major INM proteins and the associated diseases. For the purpose of this review, we briefly discuss the LEM proteins and their importance in the context of nuclear envelope repair.

## Lap2-emerin-Man1 proteins

The conserved Lap2-emerin-Man1 (LEM) family of integral INM proteins is required for tethering telomeres to the nuclear envelope and influences the integrity and structure of the nuclear envelope (Liu et al., 2003; Ulbert et al., 2006; Gonzalez et al., 2012)*.* In yeast, defects in NPC assembly are suggested to be recognized by, Heh1(Lem2) and Heh2(Man1/LEMD3) in conjunction with the ESCRT machinery (Webster et al., 2014; Webster et al., 2016b). Exposure of Lem2/Heh1 to the cytosol is a means of activation and recruitment of the nuclear envelope-specific ESCRT protein Chmp7/Chm7. Chm7 has been shown to accumulate at the sites of physically generated nuclear envelope ruptures in mammalian cells and in yeast mutants with defective nuclear envelopes (Webster et al., 2016b; Gu et al., 2017; Thaller et al., 2019a; Halfmann et al., 2019).

**FIGURE 3:**
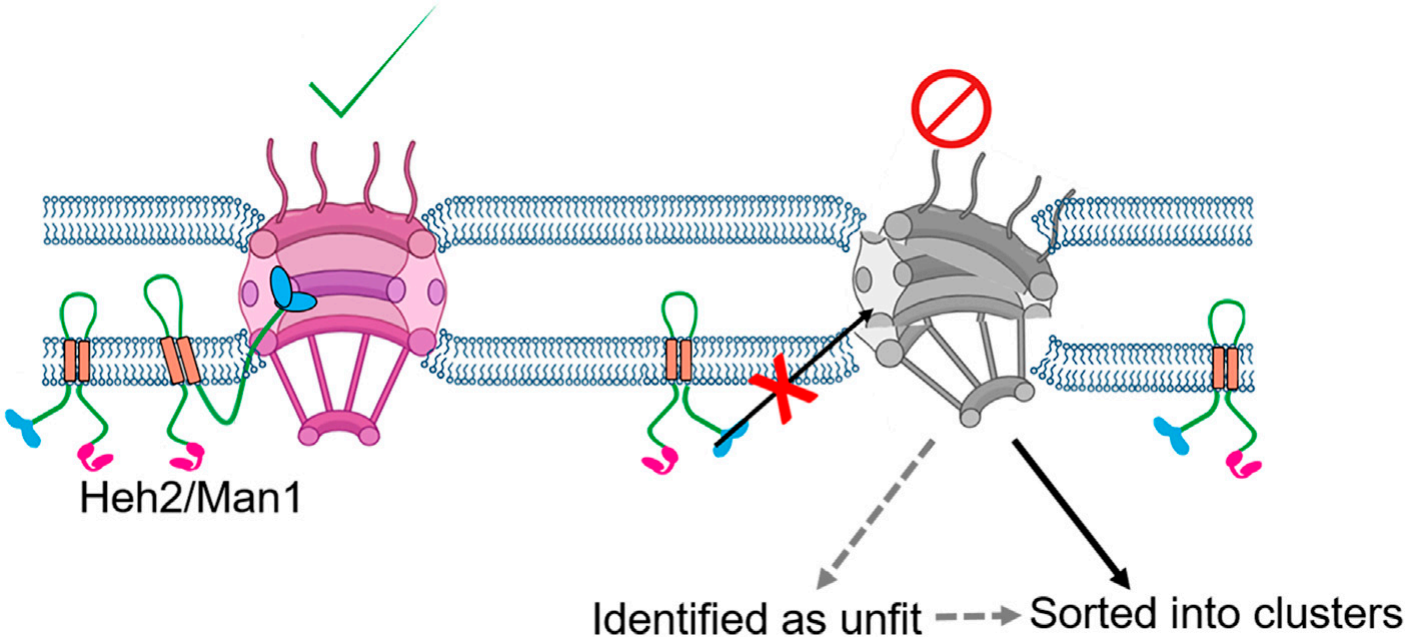
Proposed model of NPC surveillance by the LEM protein Heh2. The C-terminus winged-helix domain of Heh2 cannot bind to structurally compromised NPC and might act as a sensor of the NPC assembly state (Borah et al., 2021).

In wild type *S. cerevisiae*, Heh2-GFP associates with the NPCs, as revealed by the fluorescence microscopy and immunoaffinity assays. However, in the absence of a properly formed scaffold in mutants for the outer ring complex nups (Nup133, Nup120, Nup84), Heh2 fails to associate with those defective NPC. It is known that Heh2 binds to the NPC components *via* a winged helix domain located in its C-terminal nuclear domain (Figure 3) (Borah et al., 2021). In addition, loss of Heh2 in both *S. cerevisiae* and *S. pombe* leads to an aberrant distribution of the NPCs over the nuclear envelope. It is worth mentioning that there are only two LEM domain proteins present in both *S. cerevisiae* and *S. pombe*, ScHeh1(Src1)/SpHeh1(Lem2) and their paralogue’s ScHeh2/SpHeh2(Man1) (Barton et al., 2015). Both ScHeh1(Src1) and SpHeh1(Lem2) are believed to be derived from a common ancestor; while ScHeh2 and SpHeh2(Man1) arose independently by duplication of their respective paralogue (Rhind et al., 2011; Gonzalez et al., 2012) The evolutionary conserved molecular function of Heh2 in *S. cerevisiae* and *S. pombe*, despite their independent emergence, points to an important role in the quality control of NPCs (Borah et al., 2021).

Integral membrane proteins of the ER and nuclear envelope, Brr6, Brl1 and Apq12, which are required for lipid metabolism, link alterations in membrane fluidity to NPC biogenesis in yeast (Scarcelli et al., 2007; Lone et al., 2015; Zhang et al., 2018). The deletion of *APQ12* causes abnormal nuclear envelope morphology and defects in the assembly and distribution of NPCs as well as mRNA export (Baker et al., 2004; Scarcelli et al., 2007). Brl1 interacts with a subset of nups required for early NPC assembly, and Brr6/Brl1 clusters can recruit nups to the nuclear envelope, suggesting a role in NPC assembly (Zhang et al., 2018). Similarly, deletion of ER proteins Rtn1 and Yop1, which promote membrane curvature, has been shown to cause defects in the distribution and morphology of NPCs, and nuclear import function in *S. cerevisiae* (Dawson et al., 2009).

Intriguingly, a decline in the abundance of Heh2 and other NPC assembly-associated proteins, Apq12, Brl1, and Vps4 are strongly correlated to replicative ageing in yeast cells; a decline in the abundance of these proteins leads to an increase in misassembled NPCs at the nuclear envelope (Rempel et al., 2019).

## Nuclear envelope breakdown

Apart from assembling, scaffolding, and maintaining a functional NPC into the nuclear envelope, the other commonly faced challenge is nuclear envelope breakdown (NEBD) and its reformation in an open form of mitotic cell division. This process begins with NPC permeabilization and NE invagination in the proximity of the centrosome followed by lamin network dissolution and NE-chromatin contact detachment along with the pulling forces of mitotic spindle *via* the dynein/ dynactin complex leading to NEBD. This process of NE tearing spreads from a site distal to the centrosome ending up in complete NE fragmentation (Puhka et al., 2012) in the case of an “open mitosis” which is seen among the metazoans. In contrast,the ‘closed mitosis’ form is the most common mechanism in case of lower eukaryotes like *S. cerevisiae* and *S. pombe*. However, there are exceptions like *Cryptococcus neoformans* (Mochizuki et al., 1987), *Ustilago violaceae* (Heath, 1980) and *Ustilago maydis* (O’Donnell and McLaughlin, 1984), which undergo a different form of “open mitosis”. In the case of *U. maydis*, certain bud-specific factors activate the spindle pole body (SPB) residing in the outer membrane and lead to a dynein-dependent SPB migration into the bud and NE breakage at the point of SPB attachment (Straube et al., 2003) rather than the opposing end of NE as in higher eukaryotes. Another prevailing form of mitosis is the partial disassembly of the NE in certain higher eukaryote cell types called “semi-open” mitosis. In this type of mitosis, the NE rearrangements are minimal, allowing the loss of integrity in the region adjacent to existing centrosomes, thereby allowing microtubules to pierce through them to capture the chromosomes. A few examples of semi-open mitosis are *C. elegans* early embryos and the syncytial embryonic divisions in *Drosophila melanogaster* (Cohen-Fix and Askjaer, 2017).

To appreciate the complexity of NE rearrangements, we shall summarize the dynamics of metazoan NE remodeling during cell cycle and beyond to the best of our current knowledge in the following section.

### Nuclear envelope breakdown in open mitosis

**FIGURE 4:**
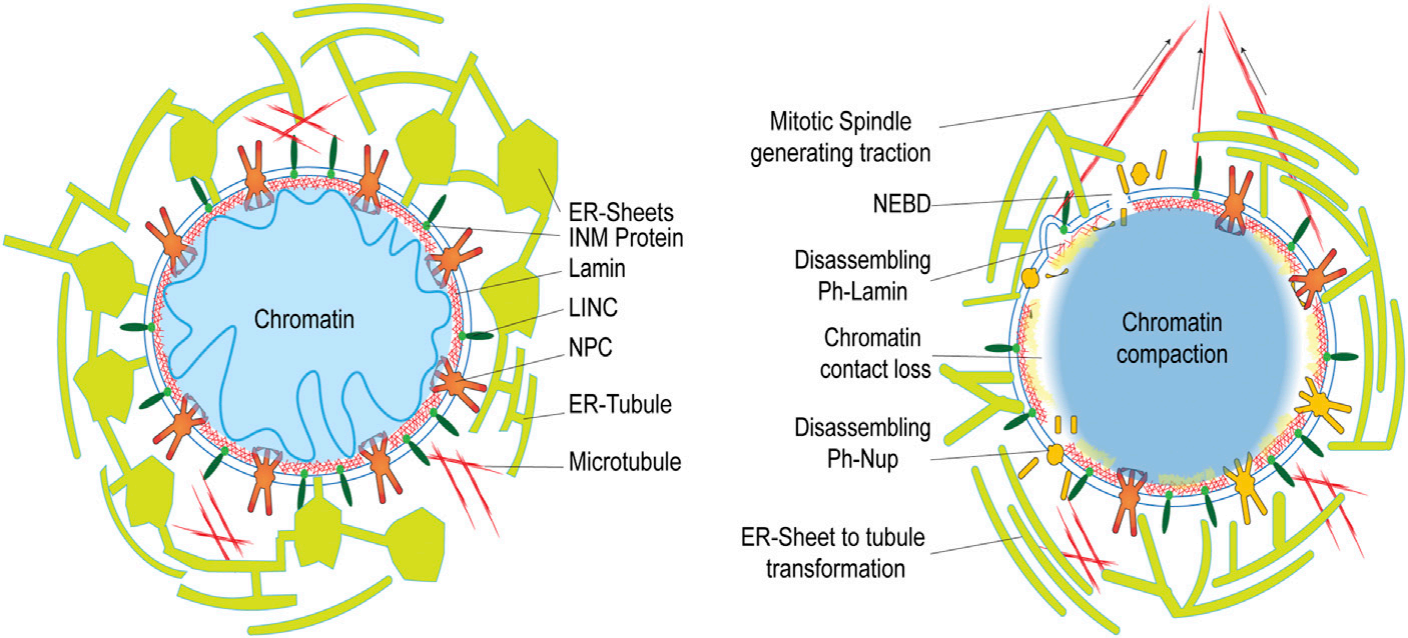
Rearrangement of the nuclear components at the onset of cell division. The left panel represents interphase NE status in a cell, while the right panel shows the status during NEBD onset during prophase in open mitosis.

At the onset of metazoan mitosis, NE breaks down during prophase. By the end of anaphase, the fragmented NE is reassembled around decondensing chromatin to establish two individual daughter nuclear compartments. Broadly, the four major factors responsible for mitotic NEBD are: 1) Phosphorylation mediated loss in protein-protein interaction, 2) Spindle microtubule generated forces acting over the envelope, 3) Endoplasmic reticulum (ER) remodeling events and, 4) Chromatin-NE tethering perturbations. All of these operate in an overlapping fashion to orchestrate the mitotic NE remodeling (Güttinger et al., 2009) (Figure 4).

Mitotic NEBD begins with gradual extraction of nups from the NPCs (Dultz et al., 2008). One of the major factors regulating this nuclear pore complex (NPC) disassembly is through nucleoporin phosphorylation (Macaulay et al., 1995; Favreau et al., 1996; Mansfeld et al., 2006; Glavy et al., 2007; Blethrow et al., 2008) which triggers NPC disintegration at the onset of prophase (Favreau et al., 1996; Beausoleil et al., 2004; Nousiainen et al., 2006; Olsen et al., 2006; Glavy et al., 2007; Blethrow et al., 2008). It is now well established that the hyper-phosphorylation of the FG nup, NUP98, by mitotic kinases like CDK1 and Nek, is the major rate-limiting step during NPC disassembly, demonstrated in HeLa cells (Laurell and Kutay, 2011). Much like NPC disassembly, the Cdk1/cyclin B mediated phosphorylation of lamins triggers nuclear lamina depolymerization (Buendia et al., 2001) favouring subsequent events in NEBD. Similarly, multiple kinases including p34^cdc2^/CyclinB, PKC, and PKA, are also known to regulate lamin B1 polymerization to assist this NEBD (Fields and Thompson, 1995). Similarly, multiple mitotic kinases, including PLK1 (Li et al., 2010), NIMA (De Souza et al., 2004), Aurora A (Hachet et al., 2007; Portier et al., 2007), ATR (Kumar et al., 2014), NEK (Laurell et al., 2011) and cyclin A2/Cdk complex (Gong et al., 2007) have been implicated in NEBD event. In general, the NE-associated proteins, including nups, lamins, and INM proteins, are phosphorylated as cells enter mitosis (Galy et al., 2008; Kutay and Hetzer, 2008), thereby interfering with multiple protein-protein interactions to initiate the process of NE dissociation. Hence, these phosphorylations act beyond their signal-transducing function in the structural rearrangement of the NE components orchestrating NEBD.

In addition to the above-mentioned NPC dissociation and lamin depolymerization, retraction of the NE and ER (Anderson and Hetzer, 2007; Kutay and Hetzer, 2008) membranes from chromatin (Güttinger et al., 2009) assisted by mitotic spindle microtubules generated force during G2/M transition helps in completing the process of NEBD (Robbins and Gonatas, 1964; Paweletz and Lang, 1988). The prevailing hypothesis, as suggested by Jan Ellenberg, is that the dynein bound to NPCs serves to attach these spindle microtubules to the NE. This allows them to create a pulling force directed from the surface of NE towards the centrosome. The increasing length and the concomitant rise in tension further lead to stretching and tearing NE around the disassembling NPCs and destabilized patches of the nuclear lamina. These events ultimately culminate in NE fragmentation (Beaudouin et al., 2002) and redistribution within the mitotic ER. Earlier, these NEBD-derived structures were considered to be a part of stable mitotic ER vesicles (Ellenberg et al., 1997; Zaal et al., 1999; Terasaki, 2000) but later *in vivo* observations supported more of a transformation of ER sheets into intact tubules during mitosis accompanied by a reduction in the membrane-bound ribosomes (Puhka et al., 2007). Further, studies with serial block-face scanning EM (SBF-SEM) based imaging has established this transformation of rough ER starting from intact or fenestrated sheets that proceeds to varying degree in different cell types to a more fenestrated or completely tubular ER network depending on the amount of ER bound ribosomes within them (Puhka et al., 2012).

The other interesting event leading to NEBD is the chromatin detachment from NE contact sites through post-translational modification (PTM) mediated disruption of anchoring proteins. Earlier, it was believed that chromatin detachment occurs passively during the breakdown process. On the contrary, recent studies in fission yeast have established an active function in regulating this event (Fernández-Álvarez and Cooper, 2017). The specific contacts between telomeric region during mitosis and centromeric regions during meiosis, are established with the Linkers of Nucleoskeleton and Cytoskeleton (LINC) complex residing within the NE. While the rest of the chromatin losses its association with the envelope to signal the initiation of localized NE disassembly (Fernández-Álvarez et al., 2016) during early prophase. In a similar study using *Schizosaccharomyces japonicas,* it was observed that the Lem2-Nur1 complex that anchors chromatin is detached from its heterochromatin contacts transiently with the aid of ESCRT-III/Vps4 machinery to allow the Lem2 enrichment over the NE tails that wraps around the spindle while re-establishing the nuclear compartment during mitotic exit (Pieper et al., 2020).

### Loss of nuclear envelope integrity beyond mitosis

**FIGURE 5:**
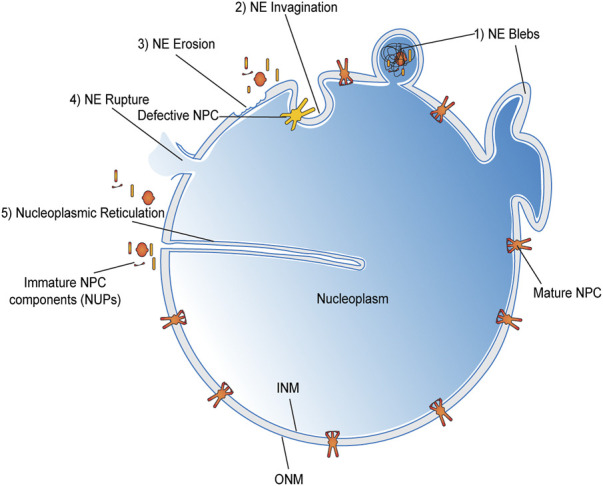
Loss of NE integrity beyond mitosis.

Multiple repair pathways operate in synchrony with the cell cycle to sustain NE integrity. The most efficient of which serves to re-establish NE between anaphase to telophase transition stage. Nevertheless, spontaneous rupture events are often documented during interphase like the defects mentioned in Figure 5, which may not have the luxury of mitotic machinery to help them repair. Although the impairments are quickly repaired, these ruptures allow ample exposure of chromosomal DNA to the cytoplasmic environment promoting DNA damage and mutagenesis supporting cancer onset. Some of the well-characterized modes of nuclear envelope rupture (NER) include (A) the transient rupture over the sites presenting structural insufficiencies in the underlying lamina (De vos et al., 2011; Hatch and Hetzer, 2014). For example, depletion of B-type lamins increased the incidence of NER. While the over-expression of Lamin B in cancer cells mitigated these rupture events (Vargas et al., 2012; Hatch et al., 2013). (B) Chromosome bridges due to flawed mitosis result in “teardrop”-shaped nuclei and defective lamin deposition around those bridges creating a structure vulnerable to NER during cell division (Maciejowski et al., 2015). (C) Micronuclei are another susceptible structures prone to NER due to their defective nuclear lamina assembly and repair mechanisms (Hatch et al., 2013; Zhang et al., 2015). Most of these micronuclei rupture due to a defective and asynchronous DNA replication during their S-phase. All these events further lead to DNA damage and chromosomal pulverization ending up in chromothripsis (Crasta et al., 2012), followed by other tumorigenic events. (D) Hyperactivated Rho GTPases (Vega and Ridley, 2008) and increased actomyosin contractility may also strain the envelope (Denais et al., 2016; Raab et al., 2016) and encourage NER to a certain degree. (E) Extra centrosomes, which are a hallmark of most cancer cells, also facilitate NER during the course of cell division (Lim et al., 2016). Now each of the above-listed factors do not act in isolation; instead they have been found to operate in tandem to promote NER in a given situation (Smoyer and Jaspersen, 2014). Apart from the ones discussed here, several other factors have also been correlated to NER in the absence of extraneous mechanical stress, like the drop in p53, p21, and Rb activity, and many more (Maciejowski et al., 2015; Yang et al., 2017) such factors in cancer cells. Yet, their mechanism of operation remains undocumented at present.

## Nuclear envelope defects contributing to cancer

As mentioned above, the loss of NE integrity either while resealing the daughter nuclei as they exit mitosis or the rupture events that occur during interphase leads to a situation where the chromatin is left exposed to various genotoxic agents residing in the cytoplasm. Hence, upon NE rupture, multiple mutations are induced in the genome that might lead to carcinogenesis when the DNA repair machinery fails to re-establish its original nucleotide sequence. Further, NE rupture has been linked to micronuclei formation, protein mislocalization, chromatin rearrangement, and genomic instability events that give rise to cancer (Vargas et al., 2012). Micronuclei are indicators of genomic instability and are a hallmark of cancer (Fenech et al., 2011). Envelope ruptures in micronuclei are irreversible and have been identified in human non-small cell lung cancer (Hatch et al., 2013). Genomic instability at the micronuclei has been shown to be protected by Chm7 and ESCRTIII by membrane repair (Willan et al., 2019). At the same time, an increased ESCRTIII accumulation in acentric micronuclei and aberrant membrane remodeling leads to membrane damage and exposure of DNA to the cytosol (Willan et al., 2019; Vietri et al., 2020). Exposure of DNA to the cytosol has been shown to activate protective proinflammatory pathways in cancer cells (Mackenzie et al., 2017; Willan et al., 2019)

Defects in NE other than NER that are documented as cancer predisposing factors like NE invagination and Nucleoplasmic reticulation (NR) can also occur in both dividing and resting cell nuclei (Malhas and Vaux, 2014; Alvarado-Kristensson and Rosselló, 2019). Since the outline of normal cells is generally smooth in appearance (Fischer, 2014), an altered NE morphology often serves as a marker to assess the tumor grade and predict its future outcome (Bussolati, 2008; Dey, 2009; Bussolati et al., 2014). In fact, one can appreciate these NE invaginations and rupture events frequently in the micronuclear structures associated with cancer cells. put together either type I involving INM alone or type II involving both the INM and ONM (Brandes et al., 1965; Clubb and Locke, 1998). In either case, the nuclear lamina or other NE-associated proteins seems to be defective and dysfunctional, which might serve as a cause or consequence of cancer (Lammerding et al., 2004; Williams and Urbé, 2007; Willis et al., 2008; Kong et al., 2012; Swift et al., 2013; Ferrera et al., 2014). Interestingly, these NE insufficiencies also aid in processes like tumor metastasis and invasion (Dey, 2009; Friedl and Alexander, 2011). A drop in lamin A/C expression has been shown to help cancer cell migration within the confined space, but in this process, it also renders such cells to be prone to physical stress that could lead to new nuclear rupture events as cancer cells invade a new site (Harada et al., 2014; Denais et al., 2016; Raab et al., 2016). Similarly, the LINC complex plays a significant role in cancer metastasis, much like the lamin proteins when they are deregulated in cancer cells (Matsumoto et al., 2015; Yajun et al., 2017; Infante et al., 2018).

In addition to these NE deformations, other nuclear lamina and nuclear membrane proteins like Lamins, Nups, etc, contribute to oncogenesis mainly due to altered chromatin dynamics as a consequence of disturbed anchoring or epigenetic hallmarks of chromatin. For example, upon silencing lamin B1, the H3K27Me3 mark representing silent chromatin becomes more dispersed in the nuclear interior (Somech et al., 2005; Camps et al., 2014). Likewise, lamin’s A/C mutations affect the heterochromatin status of a cell harboring them (Sullivan et al., 1999; McCord et al., 2013). Moreover, lamins and lamin B receptors (LBR) play a major role in maintaining a transcriptionally repressive environment around the periphery of the nucleus (Polioudaki et al., 2001; Bickmore and van Steensel, 2013). When their expression is deregulated, they activate genes once repressed and might contribute to tumorigenesis.Similarly, Lamin-associated polypeptide-2β (LAP2β) (Sikka et al., 1975; Somech et al., 2005) and Emerin (Holaska et al., 2003; Wilkinson et al., 2003) can regulate these chromatin states and have been documented to be deregulated in specific cancers. Likewise, nups like Nup98, Nup153, Nup88, TPR, and others have been established as important factors regulating chromatin transcriptional state of chromatin (Capelson et al., 2010; Liang and Hetzer, 2011; Kuhn and Capelson, 2019). Hence, an alteration in the nucleoporin-chromatin association often favors cancer onset and tumor progression (Köhler and Hurt, 2010).

## Nuclear envelope and genome organization/regulation

It is well established that the chromatin is often tethered to the nuclear periphery by means of nuclear lamina together with the nuclear envelope transmembrane proteins (NETs). This positioning is universal across cell types for certain facultative heterochromatin, but for others, it depends on the expression pattern of certain tethering proteins depending upon the state of differentiation (Parada et al., 2004). The very first of this genome organization at the nuclear periphery was reported by Rabl (1885) for centromere positioning towards the nuclear periphery and later confirmed by Cremer et al. (1982). Despite numerous investigations on centromere positioning, the tethering mechanism for such an organization remains an unsolved puzzle. Presently, multiple studies in this field have revealed quite a few NE-associated proteins in establishing such genome organisation across the nuclear periphery. Some of the established players include lamins, Lamin B receptor (LBR), and INM proteins that either directly bind chromatin or *via* chromatin-binding proteins (Czapiewski et al., 2016). Proteins like LBR and LEM-domain proteins (Lap2β, Emerin, and Man1) directly bind chromatin (Berk et al., 2013b; Nikolakaki et al., 2017), whereas NETs like Lap2β, Emerin, and Man1 could bind chromatin through its interaction with barrier-to-autointegration factor (BAF) (Zheng et al., 2000; Margalit et al., 2007; Demmerle et al., 2012; Berk et al., 2013a). Similarly, LBR binds HP1 (Amendola and van Steensel, 2014) to establish heterochromatin towards the nuclear periphery. Ultimately the importance of NE proteins in genome organization and gene expression is displayed from disorders involving mutant NETs blocking their gene regulation and chromatin organizing ability which leads to pathological outcomes like Hutchinson-Gilford Progeria, Mandibulolacral dysplasia (Cenni et al., 2018), Familial partial lipodystrophy (Bagias et al., 2020), Dunnigan-type familial partial lipodystrophy (Nabrdalik et al., 2013), Pelger-Huet anomaly (Hoffmann et al., 2002) and Emery-Dreifuss muscular dystrophy (Worman and Schirmer, 2015), where Lamins and NETs like LAP1 (Shin et al., 2013), FLH1 (Tiffin et al., 2013), SUN1, SUN2 (Meinke et al., 2014) Nesprin (Zhang et al., 2007) have been shown to be mutated and dysregulated leading to altered NE-chromatin and NE-Cytoskeletal contacts majorly impacting the genome organization and stability.

The other interesting NE component deployed as an architectural platform is the NPC, which can influence genome organization and gene expression by arbitrating the transcription factors and multiple other chromatin-associated proteins (Rowley and Corces, 2018). The NPCs serve well beyond their channeling functions by serving as reservoirs for distinct chromatin structures (Francastel et al., 2000; Galy et al., 2000; Wente, 2000; Ishii et al., 2002; Su et al., 2018). NPCs have been proposed to be the sites where transcriptional memory of genes is established by locus relocation and gene-looping events aiding in transcriptional activation of such loci (Brickner and Walter, 2004; Tan-Wong et al., 2009; Ahmed et al., 2010). In addition, NPC components can mediate repression in a variety of instances like telomere silencing (Galy et al., 2000); subtelomeric genome heterochromatization (Van de Vosse et al., 2013) Swi6 mediated (Iglesias et al., 2020) and Polycomb mediated (Gozalo et al., 2020) heterochromatin maintenance and transcriptional start site silencing *via* Polycomb repressive complex during pluripotency maintenance (Jacinto et al., 2015) where individual nucleoporin components have been found to help them establish these epigenetic states. Apart from these, recent findings have also aided us in discovering the nuances of several Nups in regulating gene expression during embryonic development (Capelson et al., 2010; Kalverda et al., 2010) and differential tissue-specific expression states as well (Raices and D’Angelo, 2017). All these put together highlight the essential role played by various nuclear envelope components in the organization and maintenance of the genome architecture. Hence maintaining the integrity of NE under the constantly challenging environment warrants dedicated repair machinery in any organism. These components of repair shall be discussed in the following sections.

## Nuclear envelope repair

One of the surprising facts from the nuclear membrane rupture analysis is that cells have a strong nuclear membrane repair mechanism during the interphase. The length of an individual rupture of the nuclear membrane is only a few minutes (de Noronha et al., 2001) but the membrane repair occurs within a few minutes to a few hours, and has been observed in many *in vitro* settings (Hatch and Hetzer, 2014; Denais et al., 2016; Raab et al., 2016; Yang et al., 2017). Remarkably, most of the cells are capable of restoring the NE integrity even after repeated NE rupture events (De vos et al., 2011; Vargas et al., 2012; Denais et al., 2016; Hatch and Hetzer, 2016; Raab et al., 2016; Robijns et al., 2016). Therefore, an efficient nuclear membrane repair is likely essential for cellular viability after rupture. Several NE assembly proteins that recruit and reseal the nuclear membrane at the end of mitosis accumulate at interphase membrane breakdown sites and promote nucleus re-compartmentalization (LaJoie and Ullman, 2017). Different types of nuclear membrane repair mechanisms have been suggested, including the attachment of ER sheets to the naked chromatin, distribution of the existing ONM, plugging by membrane recruitment, and resealing by protein complexes instead of membranes. Although there is strong evidence that multiple pathways exist, the recruitment of new ER has received the most experimental support for those documented repair. The rest need more rigorous investigations in the future to support their existence.

## Membrane repair *via* recruitment of endoplasmic reticulum and BAF/ESCRT-III machinery

The proteins recruited to rupture sites mostly overlap with those that localize telophase chromatin to the core region and play an important role in the assembly of mitotic NE. These proteins include the conserved LEM family of integral INM proteins (Lem2/Heh1 and Man1/Heh2), ESCRT machinery, BAF (barrier-to-autointegration factor), and lamin proteins (Le Berre et al., 2012; Denais et al., 2016; Penfield et al., 2018; Halfmann et al., 2019; Young et al., 2020).

Originally identified as a vacuole-sorting complex, the ESCRT machinery was subsequently recognized to mediate several more cellular processes, such as cytokinetic abscission, repair of the plasma membrane, endocytosis and secretion, assembly and budding of viral particle at the plasma membrane, and incorporation of NPC into the NE (Katzmann et al., 2002; Henne et al., 2011; Webster and Lusk, 2015; Christ et al., 2017). The ESCRT machinery mediates membrane remodeling in all of these processes by fostering membrane curvature and, in most cases, generating membrane scission. Multiple subunits constitute the ESCRT machinery, ranging from ESCRT-0 to ESCRT-III. However, resealing the nuclear membrane during NE reformation and NE repair requires only ESCRT-III members (Olmos et al., 2015; Vietri et al., 2015; Denais et al., 2016; Raab et al., 2016). The ESCRT-III complex transiently localizes to the NE in late anaphase at locations where gaps remain open. At the rupture site, the ESCRT-III complex forms a structure, twisting the membrane into a negative curvature and eventually resulting in membrane scission (Henne et al., 2011; Christ et al., 2017). VPS4B is an AAA-ATPase, which is recruited to the spiral structure and, after membrane scission, disassembles the ESCRT subunits (Henne et al., 2011; Christ et al., 2017). The ER-protein CHMP7 and the INM protein LEM2 are necessary to recruit the ESCRT-III complex at rupture sites (Vietri et al., 2015; Denais et al., 2016; Webster and Lusk, 2016). CHMP7 is typically located on the ER membrane *via* its curvature-sensitive membrane-binding domain but accumulates in the NE during NE reformation (Olmos et al., 2016). This CHMP7 initiates the assembly of the CHMP4B, ESCRT-III subunit that eventually drives nuclear membrane scission. Interaction of the spiral ESCRT-III with VPS4B is mediated by the CHMP2A subunit of ESCRT-III, which is also integrated into the spiral ESCRT-III ultimately (Effantin et al., 2013; Webster et al., 2014; Webster and Lusk, 2016).

Interestingly, LEM2 is also an equivalent of Heh1 in yeast, which has a similar feature of recruitment under NPC monitoring *via* ESCRT. CHMP7 initiates an assembly of the key ESCRT-III subunit, CHMP4B, that eventually drives the scission of the nuclear membrane (Webster and Lusk, 2016). However, the mechanisms for recruiting CHMP7 to NE-rupture sites to initiate ESCRT-III mediated NE repair are unclear. Recent work suggests that during NE reformation, LEM-domain proteins, typically found in the INM, can recruit CHMP7 to exposed sites (Gu et al., 2017). In yeast, Heh1 binds to CHM7, homolog of the mammalian CHMP7, and this complex is related to nuclear membrane sealing and pore content monitoring (Webster and Lusk, 2016). As a result, the interaction between these proteins may initiate ESCRT-III assembly and the subsequent nuclear membrane remodeling.

In addition, ATPase p97 and its cofactors UFD1 and NPl4 are important for the positioning of ESCRT-III machinery over ruptured sites (Olmos et al., 2015). The function of the p97 ATPase is unclear in this process. However, it has been reported that p97 usually recognizes ubiquitinylated membrane proteins through its UFD1 cofactor (Woodman, 2003). Thus, it is possible that ubiquitinylation of NE proteins may be involved in controlling NE sealing. Ubiquitination of Nesprin-2G has been shown to uncouple the LINC complex from the actin cytoskeleton, thereby reducing the mechanical stress and facilitating nuclear envelope sealing by ESCRT during interphase (Wallis et al., 2021).

Other than these factors, microtubules remaining around the chromatin area after spindle disassembly interferes with the NE closure during nuclear reformation (Penfield et al., 2020). The microtubule-degrading enzyme Spastin is recruited to the nuclear envelope-microtubule intersections to sever off the microtubules making way for ESCRT-III to seal the holes where microtubules cut across the reforming NE, thereby coordinating spindle disassembly and NE sealing (Vietri et al., 2015).

During mitosis, the localization of the LEM-domain containing proteins at chromatin is somewhat dependent on BAF (Haraguchi et al., 2001; Haraguchi et al., 2008). BAF is a small nuclear envelope protein that homodimerizes and cross-links with chromatin mass and also binds with nuclear LEM-domain proteins and lamin A or histones to promote the formation of a single nucleus (Jamin and Wiebe, 2015). BAF also promotes membrane resealing during NE assembly through the recruitment of LEMD2 that binds to the ESCRT-III Chmp7 protein (Olmos et al., 2016; Webster and Lusk, 2016; Gu et al., 2017; von Appen et al., 2020). Nevertheless, the DNA-and LEM-binding domains of BAF are required for its function, and the loss of the LEM-binding domain does not rescue membrane repair defects (Halfmann et al., 2019; Young et al., 2020). BAF is an important protein, presumably because its absence induces extensive multinucleation in mitosis due to the loss of cross-linking with DNA (Samwer et al., 2017). ESCRT-III mediated recruitment primarily coordinates the removal of microtubules from chromatin by sealing the resulting membrane gaps (Olmos et al., 2015; Vietri et al., 2015). Loss of either ESCRT-III or LEM2 causes defects in nuclear morphology and delays in membrane repair (von Appen et al., 2020). However, the recruitment of the ESCRT-III complex by BAF and its relevance to nuclear membrane repair is still unknown. The latest model for the repair of the nuclear membrane is close to the NE assembly at the end of mitosis. According to this model, cytoplasmic BAF accumulates rapidly, forms a complex with bare chromatin at membrane rupture sites, and initializes the recruitment of the ESCRT-III as well as the new ER membrane to patch the membrane hole and reseal the remaining membrane gaps (Denais et al., 2016; Halfmann et al., 2019). Depletion of BAF significantly reduces ER number at damage sites and impairs nucleus repair mechanisms, indicating that the surface of the exposed chromatin is initially protected by BAF (Halfmann et al., 2019).

In addition, a novel route of regulation by phase separation of the Lem2 protein has been described (von Appen et al., 2020). Lem2 protein could phase separate through a low complexity domain adjacent to the LEM domain and co-assemble with the ESCRTIII protein, Chmp7, at the nuclear envelope (von Appen et al., 2020). During nuclear reformation, Lem2 and Chmp7 form a molecular O-ring around the spindle and facilitate nuclear sealing (von Appen et al., 2020)

Recent research has shown that the membrane fusion activity of the ESCRT-III complex is limited to pores of 50 nm or less, suggesting that ESCRT-III has additional unexplained roles in membrane rupture and repair (Gatta and Carlton, 2019). Several studies indicate that membrane repair could be prevented by over-accumulation of Chmp7, ESCRT-III, or ER membranes. Additionally, the abnormal transfer of Chmp7 to the nucleus caused membrane rupture due to membrane deformation (Vietri et al., 2015). Furthermore, over-recruitment of ER sheets can obstruct membrane resealing at rupture sites, indicating that timely release of BAF from rupture sites is crucial for a successful membrane repair (Halfmann et al., 2019; Penfield et al., 2020). Similarly, the phosphorylation of BAF by VRK1 protein kinase is crucial for the detachment of BAF at rupture sites (Molitor and Traktman, 2014; Halfmann et al., 2019). Put together, these studies show that BAF/VRK1 signaling is vital for both regular NE architecture maintenance and the execution of an effective nuclear membrane repair.

## Discussion

The nuclear envelope is continually remodeled during critical cellular processes like NEBD and reformation during cell division, NPC insertion, nuclear egress of large ribonucleoproteins, and removal of defective NPCs. Our current knowledge of NE remodeling/repair is largely limited to the ESCRT machinery and the NE adaptor Heh1/Lem2, and Heh2 in surveilling the nuclear pore complex. The potential roles for other likely players, such as local lipid and membrane biosynthesis, membrane fusion proteins, and ER proteins, are still unexplored.

Recently, several studies have strongly proposed many alternative mechanisms for nuclear membrane repair. During meiosis, microtubules are recruited at the rupture site in *C. elegans*, and microtubule associated holes are sealed by ER sheets (Penfield et al., 2020). These studies indicate that the recruitment of membranes alone can restore the integrity of the nucleus after rupture. During damage, the membrane flow from the ER to the ONM is increased, which also promotes sealing gaps (Penfield et al., 2020). Remarkably, Lem2 in yeast inhibits this mechanism, indicating that activation of one repair pathway may suppress others (Kinugasa et al., 2019; Kume et al., 2019). It will be interesting in the future to define the precise role of these proteins in the context of membrane repair.

Another interesting situation where NEBD is known to occur is when a cell encounters certain viral infections. For instance, in the case of Herpesvirus infection, the nucleocapsids bud at the inner nuclear membrane which further moves into the cytosol using a nuclear egress complex (NEC). In the absence of the NEC these viruses use NEBD as an alternative egress pathway to release the virions (Grimm et al., 2012). Similarly, Parvo viral protein VP1u interacts with the Nup358, Nup214, Nup153, and Nup62, directly leading to the exposure of VP1u on the surface of the virus followed by elevated local Ca++ in the nuclear periphery and lamin phosphorylation dependent depolymerization triggering NEBD (Porwal et al., 2013). Apart from the ones reported to date, there is far more scope to discover in terms of the mechanism operated by different viruses that have the opportunity to challenge the host nuclear membrane integrity.

During cell division, telomeric and centromeric-LINC interactions control NE disassembly by creating localized concentrations of factors that confer NE disassembly. This might be an evolutionary advantage to coordinate the preparedness of chromosomes and the onset of NEBD (Fernández-Álvarez and Cooper, 2017). Yet, these factors that concentrate on the envelope are still not identified. As discussed above, an altered NE morphology serves as a marker for tumor grade assessment and to predict the prognosis (Bussolati, 2008; Bussolati et al., 2014). Most often, the rupture events are rapidly repaired by the ESCRT-dependent NE repair machinery along with DNA damage repair proteins (Denais et al., 2016; Raab et al., 2016). However, nuclear rupture could also be a unique scenario where a “fragile-nucleus” phenotype might be more vulnerable to certain novel therapeutic targets in the future (Lim et al., 2016).

Although multiple tethering components involved in NEBD have been identified thus far, this list remains vastly incomplete today. Likewise, an increase in intracellular Ca^2+^ ion concentration has also been shown to drive NEBD (Steinhardt and Alderton, 1988; Twigg et al., 1988; Kao et al., 1990), but contradicting this John Carroll lab has unequivocally demonstrated that neither global nor local Ca^2+^ ion release is necessary for NEBD in mouse embryos (FitzHarris et al., 2005). Hence, the role of Ca^2+^ ions in mammalian NEBD is still debated even though increased intracellular Ca^2+^ ion levels accelerate NEBD. So far, all these observations have only opened up more questions that remain open for future investigation.

Future research in the field will improve our fundamental understanding of nuclear envelope biology that has broad implications for human health with probable outcomes in the form of new components and mechanistic insight into nuclear envelope repair pathways. Thus providing us with a better understanding of pathologies associated with immune decline, neurodegenerative diseases, viral infections, ageing, and laminopathies. Apart from cell biology, a better understanding of nuclear biology has translational potential in the form of designing new technologies for artificial membrane fusion required for targeted drug delivery systems, *in vitro* fertilization, and protoplast fusion-like scenarios while blocking this fusion might be of value in the context of viral replication in the future days to come.
